# Is the Use of Antimicrobial Photodynamic Therapy or Systemic Antibiotics More Effective in Improving Periodontal Health When Used in Conjunction with Localised Non-Surgical Periodontal Therapy? A Systematic Review

**DOI:** 10.3390/dj7040108

**Published:** 2019-11-18

**Authors:** Animesh Pal, Sanjeev Paul, Rachel Perry, James Puryer

**Affiliations:** 1 Bristol Dental School, Lower Maudlin Street, Bristol BS1 2LY, UK; ap15666@my.bristol.ac.uk (A.P.); sp15909@my.bristol.ac.uk (S.P.); 2 University Hospitals Bristol Research and Education Centre, Upper Maudlin Street, Bristol BS2 8AE, UK; rachel.perry@bristol.ac.uk

**Keywords:** periodontitis, scaling, photodynamic, therapy, antibiotics, outcome

## Abstract

Periodontal disease can be treated in several ways. This paper reviewed whether antimicrobial photodynamic therapy (aPDT) in addition to scaling and root planing (SRP) produces improved clinical results over systemic antibiotics (ABs) in conjunction with SRP in adults with periodontitis. Studies were searched using the following electronic databases: MEDLINE, the Cochrane Database of Systematic Reviews, and the Web of Science Core Collection up to and including November 2018. Four randomized controlled trials (RCTs) were reviewed to maximise the reliability of the evidence. All participants suffered from either chronic or aggressive periodontitis and each study contained SRP as an adjunct to ABs or aPDT. To determine whether groups showed improvement after treatment, the outcome parameters chosen were probing depth (PD), clinical attachment level (CAL), and bleeding on probing (BOP). Despite finding significant improvements in all groups, the differences among groups were not consistently statistically significant. The lack of homogeneity in the results among these studies indicates that it was not possible to determine a conclusion about whether aPDT or AB as an adjunct to SRP provides better improvements in periodontitis as measured by PD, CAL, and BOP. Further studies with more similar study designs are required before firm conclusions can be deduced.

## 1. Introduction

Periodontal disease is the inflammatory process that occurs in response to chronic infection or a build-up of dental plaque around the tooth tissue. Anaerobic bacteria including primarily *Porphyromonas gingivalis*, *Tannerella forsythus*, and *Treponema denticola* cause periodontal tissue destruction, alveolar bone loss, and eventually the loss of the tooth [1]. Currently, the most widespread treatment for periodontitis involves the physical removal of plaque through debridement and/or using antimicrobial agents [2]. Initially, scaling and root planing (SRP) is carried out with the use of hand or ultrasonic instruments to debride the root surface, allowing the formation of a long junctional epithelium [3].

Clinically, it is difficult to determine the effectiveness of the debridement, especially where there has been extensive tissue loss and the periodontal pockets are deep and where root furcations are difficult to access [4]. Thus, small amounts of bacteria are likely to be left residing in the pocket with the significant effect of continual breakdown of tissue [5].

Without surgical debridement, the use of local or systemic antibiotics (ABs) can assist towards the treatment outcome. However, their use is limited to specific patients (such as those diagnosed with aggressive periodontitis) and is commonly used in practice for those who have previously had two courses of SRP with no improvement in periodontal pocket depth [6]. Furthermore, the undesirable side effects and increase in AB resistance over time have reduced the number of cases treated in this way [7].

For these reasons, it is expected that the discovery of an alternative method would be ideal to remove the periodontal bacteria from the pocket without any unwanted negative side effects. Antimicrobial photodynamic therapy (aPDT) has the potential to become a conventional therapy used in conjunction with SRP. The photosynthesiser used in aPDT is photoactivatable and binds to specific cells and is activated by a light source (a low intensity laser with a suitable wavelength) in the presence of oxygen. This produces free radicals or reactive oxygen species, primarily singlet oxygen, and causes localised photodamage cell death [8]. The aPDT has the advantage of targeting and immediately suppressing specific bacterial cells. In addition, it has a minimal risk of AB resistance and side effects, and as this is the main issue with the AB, it makes this a much safer long-term treatment option if found to have a significant effect on the periodontal disease [9]. A systematic review undertaken in 2016 [10] failed to draw any strong conclusions and the efficacy of both treatment types is unclear. Thus, it is important to compare their effectiveness to facilitate the current understanding of periodontal treatment by either AB or aPDT. As a result, the aim of this review is to determine whether the treatment of periodontitis using aPDT alongside SRP would produce an improvement in the clinical outcome compared to antibiotics as adjunct to SRP. 

### Inclusion Criteria

The PICO (Participants/Interventions/Comparisons/Outcomes) framework (Table 1) was used to direct the literary research to address our evidence summary question: “Is antimicrobial photodynamic therapy used in addition to localised non-surgical periodontal therapy more effective in improving periodontal health than systemic antibiotics (ABs) used in addition to non-surgical periodontal therapy?”.

## 2. Methods

### 2.1. Search Strategy

Searches were undertaken to identify the appropriate studies for inclusion in this review. Three databases used were: MEDLINE (via. OVID SP) (1946–2018), the Cochrane Database of Systematic Reviews (CDSR) (1996–2018), and the Web of Science Core Collection (1900–2018) (see Appendix A for details on the search). Only studies written in English were considered for inclusion.

### 2.2. Additional Searches

In addition to searching the databases above, handsearching into “Google Scholar” and the “Google Search Engine” was conducted using different combinations of the MeSH (Medical Subject Headings) and keyword searches used in the three databases. However, only papers that had previously been found in the search using the other three databases were found. 

### 2.3. Search Criteria

The selection criteria were decided upon through thorough discussion and research. 

### 2.4. Types of Study

Randomised control trials (RCTs) were included. They are the strongest in the hierarchy of evidence as causal inferences can be made, and concealment allocation and randomization avoid allocation bias and unbiased distribution of confounders. It was decided to keep controlled/comparative clinical trials within this review, as their double-blind methodology helps eliminate any bias. In vitro studies were to be excluded due to the potential for results not being reproduced within the clinical environment. Case series studies were omitted due to the lack of a control group. 

### 2.5. Types of Participants

All patients, of either gender, had to be diagnosed with aggressive or chronic periodontitis and aged 18 or over. Chronic periodontitis cases usually have an abundance of plaque and calculus which match with the amount of periodontal destruction. Aggressive periodontitis is characterised by a rapid rate of disease progression, absence of any systemic involvement, and familial aggregation of cases [11].

### 2.6. Types of Intervention

All patients had to have received SRP with the control group treated with AB and a test group treated with aPDT. Therefore, studies without SRP were excluded. Only diode lasers had to have been used in the aPDT. 

### 2.7. Types of Outcome Measure

Primary and secondary outcomes measuring probing depth (PD) and clinical attachment levels (CAL) were included. Probing depth and CAL are two of the most important indicators of periodontal health. 

Primary outcome measures:

- Probing depth changes.

Secondary outcome measures:

- Clinical attachment level changes;
- Bleeding on probing changes.

### 2.8. Search Outcome and Screening

**Figure 1:**
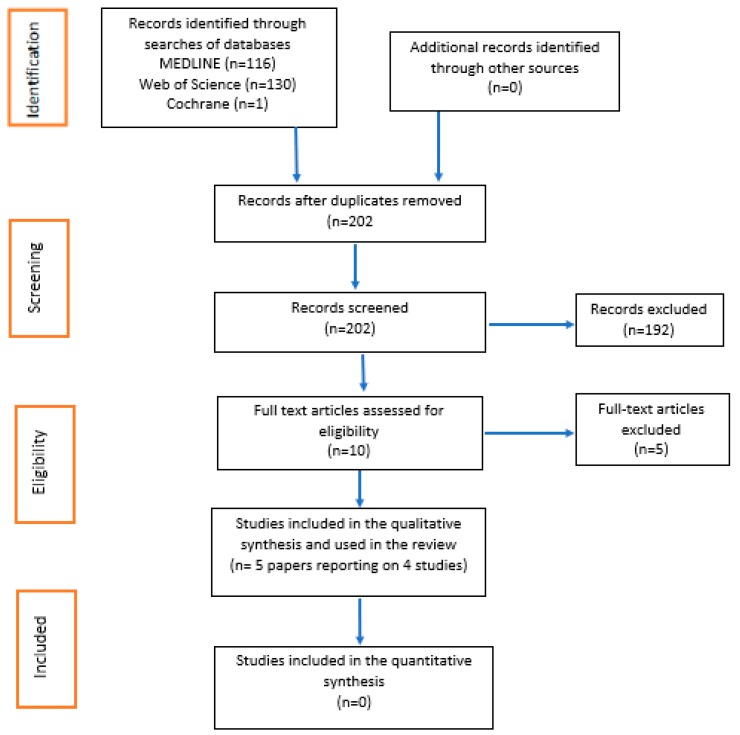
PRISMA flow diagram.

The MEDLINE, Web of Science, and Cochrane database searches completed the initial identification phase as seen in the PRISMA flow diagram (Figure 1). The first step was to remove the duplicated results from the database results. The screening phase was divided and conducted by two authors (S.P.) and (A.P.). The screening method involved reading the Title and Abstract of each paper and including them in the next phase (eligibility) if they appeared to address the designed PICO framework. For any results for which acceptability was uncertain, the second screener was consulted. If the Title or Abstract were vague, the full article was read at that point to minimise risks of excluding an eligible and appraisable paper. In the eligibility phase, both authors engaged in full readings of every paper, and using the chosen inclusion and exclusion criteria, decided which of the papers should be qualitatively analysed. Any papers that both authors deemed suitable for inclusion were automatically agreed on. For any papers where only one author believed it should be included or excluded, a decision was made upon re-reading and further discussion. 

## 3. Results

### 3.1. Search Outcome and Screening

The MEDLINE, Web of Science, and Cochrane database searches resulted in 116, 130, and 1 articles, respectively, which completed the initial identification phase of the PRISMA flow diagram. The deletion of duplicate papers resulted in 202 results. The initial screening phase reduced the number of studies to 10 that required full-text reading. The eligibility phase resulted in 5 papers reporting on 4 studies for qualitative analysis which are summarised in Table 2. The 5 full-text papers that were excluded from this review are summarised in Table A4. 

The risk of bias assessment for these four studies is shown in Table 3.

All four studies were assessed as having a low risk of bias for random sequence generation and blinding of outcome assessors [12,13,14,15,16]. Two studies were assessed as having a low risk of bias for allocation concealment [12,16], whilst this was unclear in two studies [13,14,15]. Blinding of participants and personnel was assessed as having a low risk of bias in two studies [12,16], high risk in one study [15] and was unclear in the fourth [13,14]. Incomplete data outcome was assessed as having low risk of bias in two studies [12,13,14], high risk in one study [16] and was unclear in the fourth [14]. Selective outcome reporting was assessed as having high risk of bias in two studies [12,15] and was unclear in two studies [13,14,16]. All four studies were assessed as having a low risk of bias for any other bias. The most recent 2018 study [12] scored a low risk on the most risk of bias criteria implying that this study had the least bias overall.

The four included studies in this review resulted in five papers published between 2013 and 2018 [12,13,14,15,16]. The four studies were undertaken in Brazil, Switzerland, Poland and Germany. The reviews included participants suffering from aggressive periodontitis (*n* = 3) and chronic periodontitis (*n* = 2).

A meta-analysis of the results was not conducted due to the limited number of RCTs available, the variation in interventions implemented, and the heterogeneity of outcomes reported.

### 3.2. Pocket Depth

Andere et al. (2018) [12] indicated that all therapies had a statistically significant decrease in pocket depth at three months after treatment versus baseline. However, over 3 months there was no significant difference between the SRP + aPDT and SRP group pocket depths. Over 6 months, group 3 (UPD + Clarithromycin) and 4 (UPD + Clarithromycin + aPDT) had a statistically significant higher decrease in probing depths compared to groups 1 (SRP) and 2 (SRP + aPDT) (*p* < 0.05). This decrease in pocket depth supports an earlier study by Theodoro et al. [16]. However, in contrast to Andere et al. [12], this study [16] did not find a difference in the pocket depth reduction between aPDT and antibiotics.

An earlier study by Arweiler et al. [13] in 2013 contradicts Theodoro et al. [16] regarding pocket depth results. However, it mirrors Andere et al. [12] by concluding both AB and aPDT led to significant decreases in pocket depth, and that AB had a significantly higher reduction in pocket depth than aPDT 3 months after treatment. Arweiler et al. [13] then recorded results 6 months after treatment, and this then formed a second paper [14]. These findings were in line with the three-month results, indicating that both AB and aPDT significantly decreased pocket depth, and that AB had a significantly higher reduction in pocket depth than AB.

Tabenski et al. [15] support Theodoro’s [17] findings of a reduction in pocket depth in both the aPDT and AB groups, however, 12 months after baseline. While the AB group had greater pocket depth reduction than the aPDT group, no differences were found (*p* > 0.05).

### 3.3. Clinical Attachment Loss

Andere et al. (2018) [12] observed a significant decrease in CAL in all therapy groups, although the differences in CAL between the SRP + AB and SRP + aPDT groups were not significant. Only the SRP + AB + aPDT group’s CAL reduction was significantly more than the other therapies. However, as this review is comparing SRP + AB and SRP + aPDT, this result was not valid. The results for CAL contradict the results of Theodoro et al. [16] which found that, while all therapies caused significant decreases in CAL, the SRP + aPDT decreased CAL significantly more than the SRP + AB group for intermediate pockets (≤7 mm at baseline) although not for deep pockets (>7 mm) [16].

Andere et al. [12] also support the studies by Arweiler et al. [13,14], where both the SRP + AB and SRP + aPDT groups had a significant decrease in CAL, and the SRP + AB group had a significantly greater reduction in CAL comparatively. Finally, Tabenski et al. [15] support the findings that the SRP + aPDT and SRP + AB groups both significantly reduced CAL. Whereas, in contradiction to the previous three studies [13,14,16], it did not demonstrate a between-group difference [15].

### 3.4. Bleeding on Probing

At 6 months, Andere et al. [12] demonstrated a statistically significant BOP reduction compared to baseline values. While Group 3 and 4 (the AB groups) showed less BOP compared to Groups 1 and 2, the differences were not statistically significant. This was mirrored by Theodoro et al. [16], who similarly concluded the SRP + AB and SRP + aPDT treatment groups had significantly lower BOP, although the differences among them were not significant. This is also reflected in the remaining three studies. Although significant decreases in BOP in both the SRP + AB and SRP + aPDT groups were found, the differences among them were not significant [13,14,15].

## 4. Discussion

### 4.1. Summary of Findings

The aim of this systematic review was to assess if aPDT + SRP was more effective in improving periodontal health (measured using PD, CAL, and BOP) than AB + SRP. A similar review was previously published in 2016 [10]. However, to our knowledge at the time of writing, this review is the first to include more recent studies published in 2017 [16] and 2018 [12].

In all four studies, both treatments (SRP + aPDT/UPD + AB) had a statistically significant decrease in all outcomes, reflected in two further studies [17,18].

For PD reduction, two out of the four studies concluded UPD + AB as significantly more effective than SRP + aPDT [12,13,14]. The other two studies concluded no difference in effectiveness [15,16], supporting an earlier study [19]. However, their inclusion of diabetic patients limits the scope of valid comparison. There was little consensus as to the effect of the two modalities on CAL. One study found aPDT + SRP to be significantly better in intermediate pockets and no difference among the treatment groups for deep pockets [16].

None of the studies concluded that one treatment modality was significantly better for all three outcomes. No two studies had the same result for all three outcomes, signifying a fundamental contradiction throughout the results. This means that our review cannot definitively conclude that either adjunct (aPDT/AB) is more effective than the other. The only consistent result was that none of the studies showed any significant differences among the treatment groups for BOP reduction. While this suggests that SRP + AB/UPD + aPDT are equally effective, this is only one periodontal health outcome measure. Without statistically significant PD and CAL reduction, such a conclusion is weak.

### 4.2. Strengths and Limitations

Inclusion criteria within chosen studies may have introduced bias making their results less generalisable or valid. For example, one study included both non-smoking and smoking patients [15]. Although we excluded studies that only used smoking patients, in the future we recommend excluding studies with any smoking patients.

Only two of the four studies gave oral hygiene instruction (OHI) to the sample before the treatment began, and none of the studies recorded the patient’s oral hygiene prior to treatment. Proficient OHI is necessary for any periodontal treatment to be successful by influencing calculus/plaque removal, and without it, periodontal treatment success is compromised. Lack of control over this factor in two out of the four studies questions the reliability/validity of their results. Patient demographics may have limited the studies’ generalisability as well. For example, one study used 35-year-olds or less from Sao Jose, Brazil [12], whereas another used 30 to 70-year-olds from Araçatuba (Brazil) [16]. The bio-physiological/cultural/behavioural differences among countries limit both studies’ generalisability to populations in other countries. The results of one study may be generalisable to a greater population as a wider age range was used [16] whilst another may only be valid for patients under 35 years old [12].

One study did not mention the examiner calibration; therefore, we have no quantitative measure of the precision/reliability/reproducibility of their outcome measurements [13,14]. Not accounting for the subjectivity of periodontal assessments, this may have introduced assessor bias and reduced assessment reliability. This limits the reproducibility of the conclusions drawn within this study.

Three studies [12,13,14,16] had more subjects than the minimum determined by power calculations, giving narrow confidence intervals, higher statistical power, and a possible reduction in chance results. One study used a 1.5 mm standard deviation, 0.05 significance level, and a statistical power of 0.8 to determine 17 as the minimum subject number to detect significance [15]. However, this study did not manage to recruit enough participants and so was underpowered. Loss to follow-up bias is important as those who fail to complete the study are more likely to have a different prognosis [20]. One study lost seven out of thirty-four subjects to follow-up [16]. As there was no intention-to-treat analysis [21], the loss to follow-up may have affected the power calculation to increase the likelihood of chance results.

Variation in the aPDT parameters among the studies was a key limitation. All studies had discrepancy in power density, photosynthesiser concentration, and fibre diameter, which can affect power/energy release and, therefore, efficacy [10].

The aPDT application numbers varied between one and four in the studies which could have influenced its efficacy/outcome measure improvements. For example, although one study found that SRP + AB improved PD significantly more than SRP + aPDT, only one aPDT application was used [12]. However, another study used three applications and found no significant difference among treatment groups [16], whilst another study found that three applications of aPDT enhanced clinical and microbiological outcomes [22], supporting an earlier study which found improved results with five aPDT applications [23]. This shows that increasing the number of aPDT applications improves its efficacy. Moreover, there was insufficient evidence on the optimum application number. Therefore, comparison validity among aPDT/AB was compromised as the results were affected by the bias of aPDT not being optimally given as opposed to being clinically less effective.

The inconsistency in antibiotic drugs/doses among the studies introduced bias. For example, one study concluded no significant differences between aPDT + SRP and AB + SRP effectiveness [15]; however, another found AB + UPD to be more effective [16]. This latter study’s results may be due to the use of amoxicillin + metronidazole rather than minocycline. Therefore, the differences in the results among the studies can be attributed to antibiotic type rather than the antibiotic efficacy as a better/worse therapy.

The selected studies had participants with either chronic periodontitis or aggressive periodontitis. Their differing pathophysiologies and, therefore, periodontal treatment (AB/aPDT) response [24] makes the result comparisons among patient types less valid. For example, one study’s conclusion that there was no significant difference among treatment groups for PD was confined to chronic periodontitis patients; however, another study [12] concluded SRP + AB was significantly more effective.

Another fundamental limitation in our review was that the inclusion criteria did not specify which aPDT, AB or periodontitis type/parameters were to be included. However, further RCTs with the same aPDT/AB/periodontitis parameters are recommended to increase the reliability/validity of the results, from which clearer conclusions can be made.

### 4.3. Clinical Implications

Despite the new published studies, this review reflects the conclusion of Akram et al.’s 2016 review [10] that it is not possible to determine which SRP adjunct, aPDT or AB, is more clinically effective in improving periodontal health. None of the studies in this current review concluded that either SRP + AB or SRP + aPDT was more effective than the other in reducing PD, CAL, and BOP. The results were highly contradictory. It was not possible to conclude that either treatment group had significantly better results for any outcome across all studies. Comparisons among the studies were limited by study design factors which may have affected their reliability, validity, reproducibility, and generalisability.

### 4.4. Suggestions for Future Research

There are published systematic reviews based on extensive studies that focus on the effectiveness of aPDT + SRP/SRP + AB individually. However, there is a lack of research directly comparing their effectiveness. Moreover, there is a need for further RCTs with the same aPDT/AB/periodontitis types in order to increase the comparison reliability/validity. Quantitative analysis should be included in future reviews.

## 5. Conclusions

No clear conclusion of the superior effectiveness of aPDT + SRP/SRP + AB for any outcome measure can be reached, and this strongly reflects an earlier systematic review [10]. This review highlights the need for further studies comparing aPDT + SRP/AB + SRP effectiveness using controlled parameters. This will hopefully give periodontists and dental clinicians a definitive answer as to which treatment modality, combined with non-surgical periodontal therapy, is most effective at improving periodontal health.

## Figures and Tables

**Table 1:** The PICO (Participants/Interventions/Comparisons/Outcomes) framework.

Patient/Population/Problem	Adult (>18) Patients with Periodontitis
Intervention	Photodynamic Therapy (aPDT)
Comparison	Systemic Antibiotics (ABs)
Outcome	Periodontal Pocket Depth (PD) Reduction

**Table 2:** A summary of the five papers reporting on four studies included in the review.

1st Author, Year, and Country	Total (*n*), Intervention (I), Control (c)	Patient Population, Baseline Clinical Characteristics, Demographic (Mean (SD)) or *n* Unless Otherwise Stated	Intervention	Comparator	Results
Andere et al. (2018) [12]	*N* = 36 I1 = 9 I2 = 9 I3 = 9 C = 9	**Overall Age** <35 years old **Overall Gender** 2 males 34 females **Number of teeth** All patients had at least 20 teeth	**I1: SRP and placebo pills** 2×/day for 3 days + one application of aPDT (aPDT) **I2: SRP and clarithromycin** (500 mg) 2×/day for 3 days (AB) **I3: SRP and clarithromycin** (500 mg) 2×/day for 3 days and one application of aPDT (AB + aPDT)	**SRP**: scaling and placebo pills 2×/day for 3 days	Statistically significant results were found in PD, clinical attachment level, and BOP among all intervention groups compared to the baseline. **Probing Depth (PD)** At 6 months, all intervention groups exhibited reduced PD compared to the C group. **Clinical Attachment Level (CAL)** Groups AB and AB + aPDT showed a significant gain in comparison to groups SRP and aPDT (difference in clinical attachment level between baseline and 6 months: SRP = 2.3 ± 0.8 mm; aPDT = 2.4 ± 1.1 mm; AB = 3.0 ± 1.8 mm; AB + aPDT = 3.0 ± 1.0 mm). **Bleeding on probing (BOP)** BOP decreased in all groups compared to the control group. Groups AB and AB + aPDT displayed the largest decrease (difference in BOP between baseline and 6 months: SRP = 38.8%; SRP + aPDT = 33.3%; SRP + AB = 16.6%; SRP + AB + aPDT = 16.6%
Arweiler, et al. (2013, 2014) [13,14]	36 participants started, (1 loss to FU in the aPDT group). aPDT = 17 AB = 18	**Age** aPDT gp = 37.4 ± 8.0 AB gp = 34.7 ± 9.1 **Overall Gender** 12 males 23 females	**I1: SRP and AB** (375 mg of amoxicillin and 250 mg of metronidazole 3× daily) for 7 days. **I2: UPD and AB** (400 mg of metronidazole and 500 mg of amoxicillin three times a day) for 7 days (AB)	**C1**: UPD and 2 courses of aPDT **C2**: SRP and 3 periods of aPDT (two applications on the day of SRP and 1× after 7 days)	The results from baseline to 3 months indicated a statistically significant improvement within the parameters measured among both the AB and the aPDT groups. **PD** AB group produced better results overall (5.0 ± 0.8 mm to 3.2 ± 0.4 mm). aPDT had a smaller decrease (5.1 ± 0.5 mm to 4.0 ± 0.8 mm) **CAL** AB group showed greater improvements than the aPDT group (from 5.5 ± 1.1 mm to 3.9 ± 1.0 mm in comparison to 5.7 ± 0.8 mm to 4.7 ± 1.1 mm). **BOP** AB group improved from 85.7% ± 15.9% to 34.6% ± 22.8%, while a smaller improvement was seen in the aPDT group from 70.4% ± 22.4% to 37.7% ± 21.3%. After 6 months, the AB group produced an improvement in the measures including PD, CAL, and BOP. Between-group comparisons were non-significant.
Tabenski et al. (2017) [15]	**I: 15** **C1: 15** **C2: 15**	**Median Age** aPDT + SRP gp = 54 Minocycline + SRP gp = 56 SRP alone gp = 57 **Overall Gender** 21 males 24 females	I: aPDT and SRP (aPDT)	C1 (positive control): Minocycline and SRP (AB) C2: (negative control) SRP alone	Significant improvements in clinical and microbiological parameters were found for all groups after 6 weeks and 3, 6, and 12 months. The greatest improvement came from the AB group. Between-group comparisons were non-significant. Comparing baseline to 12 months, statistically significant reductions were seen in the PD (aPDT = 2 mm; AB = 3 mm; SRD = 2 mm).
Theodoro, et al. (2017) [16]	I: 17 with 3 participants loss to FU (AB). C: 17 with 4 loss to FU (aPDT) Total analysed = 27	**Age** Between 32 and 66 years old **Gender** 15 males 12 females	SRP and AB (400 mg of metronidazole and 500 mg of amoxicillin 3 times a day) for 7 days	SRP and 3 immediate episodes of aPDT	After 90 days, statistically significant improvements were seen in all the measured clinical parameters, with the AB group having the superior improvement. **PD** AB group showed a greater decrease from 3.99 ± 0.57 mm at baseline to 3.69 ± 0.38 mm after 90 days compared to 3.78 ± 0.42 mm down to 3.54 ± 0.41 mm in aPDT. **CAL** A larger improvement in the AB group (from 4.91 ± 1.20 to 4.62 ± 1.07) when compared to the aPDT group (from 4.43 ± 0.72 to 4.25 ± 0.71). **BOP** Both groups showed a decrease, but the aPDT group displayed a greater reduction (from 78.36% ± 11.22% to 59.50% ± 13.01%) compared to the AB group (from 75.04% ± 19.09% to 60.58% ± 15.95%).

SRP = Scaling and Root Planing, aPDT = Antimicrobial Photodynamic Therapy, CAL = Clinical attachment level, PD = pocket depth, BOP = bleeding on probing, FU = follow-up, Gp = group.

**Table 3:** Risk of bias for the four studies.

	Andere (2018) [12]	Theodoro (2017) [16]	Arweiler (2013 and 2014) [13,14]	Tabenski (2017) [15]
Random sequence generation	Low risk	Low risk	Low risk	Low risk
Allocation concealment	Low risk	Low risk	Unclear	Unclear
Blinding of participants and personnel	Low risk	Low risk	Unclear	High risk
Blinding of outcome assessors	Low risk	Low risk	Low risk	Low risk
Incomplete data outcome	Low risk	High risk	Low risk	Unclear
Selective outcome reporting	High risk	Unclear	Unclear	High risk
Other bias	Low risk	Low risk	Low risk	Low risk

**Figure A1:**
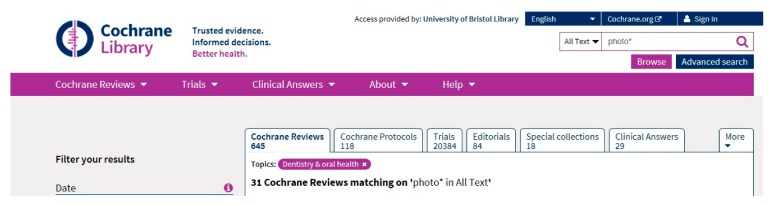
Cochrane review titles.

**Figure A2:**
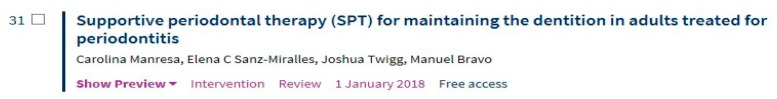
The relevant Cochrane review title.

## Appendix A

Details of the search strategy:

### Appendix A.1. MEDLINE (Via OVID SP)

Table A1 in the Appendix A summarises the search strategy used in MEDLINE to identify possible studies that could be used for this review. The four overarching concepts within our PICO framework requiring dynamic and focused search terms were photodynamic therapy, scaling and root planing, antibiotics, and periodontitis. The specific MeSH terms used by MEDLINE relating to these concepts were discovered (third column of Table A1). There was an underestimation in the variability in the terminology required. For example, clinicians commonly refer to periodontal scaling treatment as supragingival/subgingival scaling/debridement. However, in MEDLINE, the MeSH related to this treatment is: “Root Planing”, “dental scaling”, and “periodontal debridement”. Recognising this was fundamental to widening the scope of the search. However, it was important to use “scaling”, “root planing”, and “debridement” in the keyword search as the equivalent MeSH terms mentioned are not necessarily how the procedures are described clinically or in papers. Not doing so may have limited search results. Similarly, preparatory reading into the subject area highlighted this diode-laser treatment commonly referred to as “photodynamic therapy”. However, on MEDLINE it was important to recognise the MeSH for this as “photochemotherapy” which included “photodynamic therapy” alongside other descriptors. Moreover, due to the uncertainty in this terminology, we conducted the wildcard searches, photochemotherap* or photodynamic* to consider any other lexical variations; “Photo adj2 therap” was used to cover any alternative variations. Finally, a cumulative search was undertaken which included the MeSH and keyword phrases (for example, row 4 of Table A1) using the Boolean “OR” operator. This resulted in a total cumulative search of all the MeSH and keywords for the four concepts using the Boolean “AND” operator.

**Table A1:** Search strategy used for MEDLINE (Via OVID SP).

Number	Search	(MESH) Words/Phrases in This Search	Keywords in This Search	Results
1	periodontitis/or aggressive periodontitis/or chronic periodontitis/or periodontal pocket/	periodontitis, aggressive periodontitis, chronic periodontitis, periodontal pocket		23,828
2	(periodontitis or periodontal).mp.		periodontitis, periodontal	83,739
3	1 or 2			83,739
4	dental scaling/or root planing/or periodontal debridement/	dental scaling, root planing, periodontal debridement		4188
5	(“scaling” or “root planing” or “debridement”).mp.		scaling, root planing, debridement	74,702
6	Anti-Bacterial Agents/	anti-bacterial agents		307,881
7	(“antibacterial agents” or antimicrobial* or antibiotic*).mp.		antibacterial agents, antimicrobial, antibiotic	578,051
8	6 or 7			578,051
9	Photochemotherapy/	photochemotherapy		17,227
10	(Photochemotherap* or photodynamic* or (photo adj2 therap)).mp.		photochemotherap*, photodynamic*, (photo adj2 therap)	26,754
11	9 or 10			26,754
12	4 or 5			74,702
13	3 and 8 and 11 and 12			116

### Appendix A.2. Web of Science Core Collection

This database differs to MEDLINE as it is keyword searching only as opposed to subject heading and keyword searching. This made the searching strategy less complex, thus an open-ended search strategy collated as many results as possible. The four concepts within the PICO framework discussed above were covered through the keyword searches, detailed in Table A2. For example, searching “periodontitis or periodontal” covered “aggressive periodontitis, chronic periodontitis, periodontal disease and periodontal pocketing”, which was searched in the MEDLINE due the use of MeSH and keyword searches. Boolean “OR” operators were used to collate searches for each of the four concepts and then the “AND” completed the final combined search as seen in Table A2 row 6.

**Table A2:** Search strategy used for the Web of Science Core Collection.

Number	Search	(MESH) Words/Phrases in This Search	Keywords in This Search	Results
1	TOPIC: (periodontitis OR periodontal)		Periodontitis, periodontal	59,477
2	TS = (“scaling” OR “root planing” OR “debridement”)		Scaling, root planing, debridement	229,915
3	TOPIC: (“antibacterial agents” OR antimicrobial* OR antibiotic*)		antibacterial agents, antimicrobial*, antibiotic*	465,002
4	TOPIC: (photochemotherapy* OR photodynamic*)		photochemotherapy*, photodynamic*	44,680
5	1 AND 2 AND 3 AND 4			130

### Appendix A.3. Cochrane Database of Systematic Reviews (CDSR)

The Dentistry and Oral Health section has 197 (Cochrane) reviews. Therefore, the focused search strategies as required in the previous two databases were not enforced. The search strategy in this database was to enter “photo*” into the “Title Abstract Word” search tool which had a filter for “Dentistry and Oral Health”-related reviews (Table A3). This resulted in 31 Cochrane Review titles (Figure A1). A manual sift through the Title and Abstracts of these titles only found one Cochrane Review that was related to the PICO framework (Figure A2).

**Table A3:** Search strategy used for the Cochrane Database.

Number	Search	(MESH) Words/Phrases in This Search	Keywords in This Search	Results
1	“photo*” searched in Title Abstract Keyword’ filtered for Dentistry and Oral Health		photodynamic	31

**Table A4:** The five full-text studies excluded in this review.

Study and Date	Reference	Reason for Exclusion
Grzech-Leśniak et al. (2018)	[24]	Used aPDT and AB on same participants
Macedo et al. (2013)	[25]	Participants were diabetic
Ramos et al. (2016)	[18]	Participants were diabetic
Skurska et al. (2015)	[26]	Dissimilar outcome measures
Theodoro et al. (2018)	[17]	Participants were smokers
